# Effectiveness and safety of lasmiditan and rimegepant in the acute migraine treatment: an indirect real-world comparison

**DOI:** 10.1007/s10072-026-09510-6

**Published:** 2026-11-05

**Authors:** Gloria Vaghi, Luigi Francesco Iannone, Gabriele Sebastianelli, Elisa Maria Piella, Michele Corrado, Marina Romozzi, Federico De Santis, Raffaele Ornello, Francesca Pistoia, Francesco De Cesaris, Andrea Burgalassi, Gianluca Coppola, Marcello Silvestro, Innocenzo Rainero, Giorgio Dalla Volta, Danilo Antonio Montisano, Simona Guerzoni, Maria Pia Prudenzano, Davide Mascarella, Gianluca Avino, Edoardo Mampreso, Mariarosaria Valente, Stefania Battistini, Antonio Granato, Maria Albanese, Michele Trimboli, Alberto Doretti, Roberto De Icco, the Italian Headache Registry (RICe) Study Group, Cristina Tassorelli, Martina Giraudo, Francesco Casillo, Flavia Lo Castro, Rossana Allamprese, Alessia Agostinelli, Gennaro Saporito, Simona Sacco, Antonio Munafò, Alberto Chiarugi, Giovanna Carlucci, Antonio Russo, Marco Bolchini, Matteo Cortinovis, Licia Grazzi, Martino Gentile, Adriana Fallacara, Sabina Cevoli, Enrico Grassi, Catello Vollono, Sara Pez, Luca Bartole, Antonio Meo, Vittoria Carla D’Agostino, Nicola Mercuri Biagio, Eleonora Colombo, Alessandro Cocuzza

**Affiliations:** 1 Department of Brain and Behavioral Sciences, University of Pavia, Pavia, Italy; 2 Headache Science and Neurorehabilitation Unit, IRCCS Mondino Foundation, Pavia, Italy; 3 Digital and Predictive Medicine, Pharmacology and, Clinical Metabolic Toxicology-Headache Center and Drug Abuse-Laboratory of Clinical Pharmacology and Pharmacogenomics, AOU Policlinico Di Modena, Modena, Italy; 4 Department of Medico-Surgical Sciences and Biotechnologies, Polo, Pontino, Sapienza University of Rome, C.So Della Repubblica 79, 04100 Latina, Italy; 5 Department of Neurosciences “Rita Levi Montalcini”, University of Turin, Turin, Italy; 6 Fondazione Policlinico Universitario Agostino Gemelli IRCCS, Università Cattolica del Sacro Cuore, Rome, Italy; 7 Department of Biotechnological and Applied Clinical Sciences, University of L’Aquila, 67100 L’Aquila, Italy; 8 Department of Health Sciences, University of Florence, Florence, Italy; 9 Headache Center, Department of Medical, Surgical, Neurological, Metabolic and Aging Sciences, University of Campania ‘Luigi Vanvitelli’, Naples, Italy; 10 Headache Center of Clinical Neurology of Istituto Clinico Città Di Brescia, Brescia, Italy; 11 Neuroalgology Unit and Headache Center, Fondazione IRCCS Istituto Neurologico Carlo Besta, Milan, Italy; 12 L. Amaducci” Neurological Clinic, University of Bari, Bari, Italy; 13 IRCCS Istituto Delle Scienze Neurologiche Di Bologna, UOC Clinica Neurologica Metropolitana - Programma Cefalee Ed Algie Facciali, Bologna, Italy; 14 Neurology Unit, Ospedale Santo Stefano, USL Toscana Centro, Prato, Italy; 15 Headache Centre, Neurology - Euganea, Health Unit, Padua, Italy; 16 Clinical Neurology Azienda Sanitaria Friuli Centrale. Department of Medicine, University of Udine, Udine, Italy; 17 Department of Medical, Surgical and Neurological Sciences, University of Siena, Siena, Italy; 18 Headache Centre, Clinical Unit of Neurology, School of Neurology, Department of Medicine, Surgery and Health Sciences, University Hospital and Health Services, ASUGI, University of Trieste, Trieste, Italy; 19 Unit of Neurology, Department of Systems Medicine, University of Rome Tor Vergata, Rome, Italy; 20 Department of Neurosciences, AOU Renato Dulbecco, Catanzaro, Italy; 21 Department of Neurology- Laboratory of Neuroscience, IRCCS Istituto Auxologico Italiano, Milan, Italy

**Keywords:** Acute treatments, Gepants, Ditans, CGRP, Pain, Headache

## Abstract

**Aim:**

Rimegepant and lasmiditan are effective acute migraine treatments, but real-world head-to-head comparative data are lacking. We aimed to compare their effectiveness and tolerability in clinical practice.

**Methods:**

This post-hoc study indirectly compared two prospective, multicenter, real-world studies (GAINER and DART). Participants treated up to four migraine attacks with rimegepant 75 mg orally disintegrating tablets or lasmiditan 50 or 100 mg. Data were collected using an electronic diary every 30 min up to 2 hours after dosing. The co-primary outcomes were 2-hour pain freedom and incidence of adverse events (AEs) comparing rimegepant with lasmiditan in the first treated attack.

**Results:**

The overall cohort included 249 individuals, 58 treated with lasmiditan and 191 with rimegepant (81.1% females, age 45.8 [43.4–48.1] years, 9.6 [8.3 – 10.8] monthly migraine days, 26.9% with chronic migraine). Pain freedom 2-hour post dose in the first treated attack was comparable between rimegepant and lasmiditan (43.5%, n = 83 vs. 32.8%, n = 19, p = 0.171). The relative risk (RR) of achieving 2-hour pain freedom with lasmiditan versus rimegepant was 0.75 [0.50 – 1.28] (p = 0.169). The percentage of participants who reported at least one AE in the first treated attack was higher with lasmiditan than with rimegepant (53.4% vs. 11.5%; RR 4.64 [2.93–7.35], p < 0.001).

**Conclusion:**

Lasmiditan and rimegepant demonstrated comparable effectiveness, although more participants reported AEs after lasmiditan intake. Nonetheless, this indirect comparison warrants further confirmation.

**Trial registration:**

GAINER registration at www.clinicaltrial.gov: NCT05903027; DART registration at www.clinicaltrial.gov: NCT05903040.

**Supplementary Information:**

The online version contains supplementary material available at https://doi.org/10.1007/s10072-026-09510-6.

## Introduction

Recent advances in migraine management have been driven by the introduction of novel acute and preventive therapies that reshaped the treatment landscape in real-world clinical practice. Until a few years ago, acute migraine treatment relied primarily on nonsteroidal anti-inflammatory drugs, acetaminophen, and triptans, with the latter being the only migraine-specific drugs [1]. More recently, the introduction of additional migraine-specific therapies, namely rimegepant and lasmiditan, has expanded the therapeutic options for the acute migraine treatment. These therapies were developed to overcome the limitations of existing treatments, including incomplete effectiveness, contraindication and poor tolerability [2]. By expanding the therapeutic armamentarium, they have the potential to improve acute migraine management, thereby reducing disability and the risk of migraine chronification and medication overuse headache [3].

Lasmiditan is a selective serotonin 5HT_1F_ receptor agonist that ultimately inhibits calcitonin gene related peptide (CGRP) release. The 5HT_1F_ receptor is expressed in key central and peripheral neural structures of the trigemino-vascular system, including the cortex, thalamus, hypothalamus, trigeminal nucleus caudalis and trigeminal ganglion [4]. Lasmiditan’s lipophilic nature enables it to cross the blood–brain barrier, potentially contributing to central adverse events such as dizziness, paresthesia, nausea, and fatigue, and resulting in a restriction on driving and operating heavy machinery in the 8 hours after treatment [5]. The recommended dose is 100 mg, with the option to increase or decrease the dose based on effectiveness and tolerability, up to a maximum daily dose of 200 mg. Unlike triptans, lasmiditan does not activate 5-HT_1B_ receptors, and therefore does not induce vasoconstriction [4, 6]. Although it represented a valuable therapeutic alternative for individuals with contraindications to triptans, it has recently been voluntarily discontinued worldwide for reasons related to post-marketing requirements and unrelated to safety, efficacy, or quality concerns [7].

Rimegepant is an oral CGRP receptor antagonist that binds the CGRP receptor with high affinity and belongs to the gepants family. For the acute treatment of migraine, rimegepant is administered as a 75 mg orally disintegrating tablet, with a maximum recommended dose of one tablet a day. Real-world evidence supports its effectiveness and favourable tolerability profile, with fatigue, nausea and somnolence as main reported adverse events (AEs) [8]. Although the long-term consequences of CGRP receptor blockade remain to be fully established, current prescribing information does not list cardiovascular disease as a contraindication to gepant therapy [9].

Both rimegepant and lasmiditan demonstrated efficacy in the acute treatment of migraine in randomized, placebo-controlled trials (RCTs) [10–16], and in open label studies [17–20]. Building on these findings, the International Headache Society (IHS) guidelines and practical recommendations strongly support the use of both lasmiditan and rimegepant for acute migraine treatment, particularly in individuals with contraindications to triptans, such as prior stroke, cardiovascular diseases, uncontrolled hypertension, or in individuals older than 65 years [1, 21]. Real-world evidence further supported the effectiveness and tolerability of these agents [22–25].

Comparative evidence between rimegepant and lasmiditan is currently limited to indirect comparisons and network meta-analyses of RCTs [26–29]. These analyses suggest slightly higher rates of 2-hour pain freedom with lasmiditan, albeit with a greater incidence of adverse events compared with rimegepant [26–29]. However, whether these findings translate into routine clinical practice remains unknown, as no comparative analyses based on real-world data have been published to date. We previously conducted two multicentric real-world studies designed to evaluate the effectiveness and tolerability of rimegepant (GAINER) and lasmiditan (DART) using harmonized methodologies for data collection and outcomes [8, 30].

To address the current lack of comparative real-world evidence and assess their effectiveness and tolerability in routine clinical practice, we performed an indirect comparison of rimegepant and lasmiditan using data from GAINER and DART.

## Methods

### Study design and variables collected

This is a post-hoc indirect comparison of two prospective, Italian, multicentric, real-world studies: GAINER (NCT05903027) and DART (NCT05903040). They were approved as part of the *Registro Italiano Cefalee (RICe)* study by the local Ethics committee (Studio RICe, 14591_oss CEAVC Studio RICe, 14591_oss and subsequent amendments). Complete details about the RICe study are provided elsewhere [8, 31]. The list of all centres participating in the study is provided in the Supplementary Table 1. All participants signed a written informed consent before entering the study.

Data were collected by means of an online open database Research Electronic Data Capture (REDCap) on the *Empedocle* electronic platform (developed for the RICe study).

At the time of the study, neither lasmiditan nor rimegepant were subsidized by the Italian National Health Service, and participants received the drugs free of charge from the participating centers or purchased them from the community pharmacies. The prescribing headache specialist selected either rimegepant or lasmiditan according to the patient's characteristics and individual clinical judgment, reflecting routine clinical practice.

Participants were instructed to treat up to four migraine attacks with rimegepant 75 mg orally disintegrating tablet or lasmiditan 50 or 100 mg. Both studies shared the same inclusion and exclusion criteria.

Inclusion criteria were:

i) individuals aged 18 years or older;
ii) diagnosis of migraine without aura, migraine with aura, or chronic migraine (CM) according to the third edition of the international classification of headache disorders (ICHD-3) [32];
iii) at least 3 monthly migraine days (MMDs) in the 3 months preceding enrolment;
iv) expected ability to comply with study procedures;
v) availability of headache diaries covering at least 1 month before enrollment.

Exclusion criteria were:

i) any contraindications to lasmiditan or rimegepant;
ii) concomitant diagnoses of medical diseases and/or comorbidities that could interfere with the study according to investigators, including pregnancy and breastfeeding.

At baseline, headache specialists collected detailed demographic and clinical data, including headache diagnosis, accompanying symptoms, duration and severity of attacks, preventive treatments, monthly headache days (MHDs), MMDs, number of acute drugs per month, and number of days per month with at least one acute drug intake. Finally, Headache Impact Test (HIT-6) [33]and Migraine Disability Assessment (MIDAS) [34] questionnaires were collected.

According to the IHS guidelines for studies on the acute treatments of migraine attacks, a headache day was defined as any day on which a participant recorded any type of headache, while a MMD was defined as any day of headache with migraine characteristics or use of triptans [35]. 

Participants were instructed to take the study medication at their discretion, according to their usual practice, and were allowed to use rescue medication from 120 minutes after rimegepant or lasmiditan intake. They were instructed to report the following information on a specific eDiary: timing of headache onset, timing of drug intake, pain intensity at the time of drug intake (4-point Likert scale: 0 = no pain, 1 = mild, 2 = moderate, 3 = severe), functional disability assessed using the four-point Functional Disability Scale (FDS), accompanying symptoms, and the most bothersome symptom (MBS) with related intensity. The same variables were recorded at 30, 60, 90, and 120 minutes after drug intake. Adverse events (AEs) and subjective tolerability (from very bad to excellent) were collected as well.

### Outcomes

The co-primary outcomes were: i) the proportion of participants achieving 2-hour pain freedom in the first-treated attack, ii) the incidence of AEs in the first-treated attack.

Secondary outcomes included the indirect comparison of:

i) the proportion of participants achieving 2-hour pain freedom in all-treated attacks;
ii) the incidence of AEs in all treated attacks;
iii) the number needed to treat (NNT) for achieving 2-hour pain-freedom;
iv) the number-needed-to-harm (NNH) for occurrence of at least one AE​;
v) the proportion of participants achieving pain relief at 2-hour post dose (defined as decrease in pain intensity from moderate-severe to mild or no pain without intake of rescue medication);
vi) the proportion of participants achieving freedom from the MBS at 2-hour post dose.

### Statistical analysis

This is a post hoc analysis of two previously conducted studies. Sample size calculation was separately performed for the GAINER and DART studies [8, 30].

Normality test by means of the Shapiro–Wilk test proved the non-normality of several variables. Thus, statistical analyses were conducted with non-parametric tests. We reported mean [95% confidence interval] for continuous variables and number (percentage) for categorical data. Univariate analyses were performed with Mann–Whitney or Kruskal–Wallis tests for independent groups comparison, and chi-square test for categorical variables. Cochran Q test was adopted to assess significant modification of pain freedom over time. We also performed an indirect comparison between effectiveness and tolerability of lasmiditan and rimegepant through the assessment of the NNT for 2-hour pain-freedom, and the NNH for occurrence of at least one AE. NNT and NNH were rounded up to whole numbers. No imputation was performed for missing data. All statistical tests were two-tailed, and a p-value < 0.05 was considered statistically significant. Bonferroni correction was applied for multiple comparisons where appropriate. All data were analysed using SPSS software version 26.0 (IBM Corp. SPSS Statistics, Armonk, NY, USA), while MedCalc software was used for the NNT and NNH analysis.

## Results

### Study population

The final cohort included 249 individuals (81.1% females, mean age 45.8 [95% CI 43.4–48.1] years, disease duration 29.7 [25.1–34.3] years), 58 participants from the DART study and 191 from the GAINER study.

Diagnosis at enrolment was episodic migraine (EM) for 73.1% of participants (n = 182/249), and CM for 26.9% (n = 67/249), of whom 62.7% (n = 42/67) had a concomitant diagnosis of medication overuse headache (MOH). Overall, at baseline participants reported 11.6 [10.2 − 13.1] MHDs, 9.6 [8.3–10.8] MMDs, with 11.1 [9.1–13.0] monthly days of acute drugs intake and 14.4 [8.4–20.3] monthly doses of acute drugs intake. The majority of participants were receiving ongoing preventive treatment (72.3%, n = 180).

As detailed in Table 1, the two populations were comparable in terms of migraine features, drug intake and related disability. Compared to the GAINER cohort, participants treated with lasmiditan presented a higher number of previously failed classes of preventive treatments (DART: 3.2 [2.6–3.8] vs. GAINER: 2.4 [2.0–2.6], p = 0.013) and were more likely to be receiving anticonvulsants as ongoing preventive treatment (DART: 19% vs. GAINER: 7.3%, p = 0.022).

**Table 1:** Demographic and clinical characteristics of the study population

	Total		RIMEGEPANT		LASMIDITAN	*p*-value
Number of participants	**249**		**191**		**58**	-
Age (years)	45.8 [43.4–48.1]		44.8 [43.2—46.5]		49.0 [45.2–52.9]	0.060
Females, n. (%)	202 (81.1%)		153 (80.1%)		49 (84.5%)	0.567
Aura, n. (%)	28 (11.2%)		19 (9.9%)		9 (15.5%)	0.242
Chronic migraine, n. (%)	67 (26.9%)		53 (27.7%)		14 (24.1%)	0.735
Medication overuse, n. (%)	42 (16.9%)		35 (18.3%)		7 (12.1%)	0.320
Disease duration (years)	29.7 [25.1–34.3]		28.4 [25.0–31.8]		33.9 [26.7–41.1]	0.345
Duration of chronic migraine (years)	15.4 [10.5–20.3]		15.5 [10.9–20.1]		14.9 [9.2–20.7]	0.180
Monthly headache days	11.6 [10.2–13.1]		11.9 [10.8–13.1]		10.7 [8.5–12.9]	0.122
Monthly migraine days	9.6 [8.3–10.8]		9.6 [8.9–10.9]		9.4 [7.4–11.3]	0.261
Days of acute drugs intake/month	11.1 [9.1–13.0]		11.4 [9.8–12.9]		9.9 [7.1–12.9]	0.195
Doses of acute drugs/month	14.4 [8.4–20.3]		14.9 [8.6–21.3]		12.6 [8.3–16.9]	0.375
Duration of migraine attack (hours)	33.8 [26.9–40.6]		35.4 [28.7–42.2]		28.5 [21.3–35.6]	0.954
MIDAS	49.4 [36.2–62.7]		51.4 [38.3–64.4] (n = 164)		43.0 [29.2–56.7] (n = 91)	0.301
HIT-6	61.2 [57.6–64.9]		60.6 [56.6–64.6] (n = 111)		63.2 [61.0–65.3] (n = 46)	0.204
Previously failed preventive treatments						
Classes of preventive treatment	2.6 [2.2–3.0]		2.4 [2.0–2.6]		3.2 [2.6–3.8]	**0.013**
Anti-CGRP mAbs	23 (9.2%)		19 (9.9%)		4 (6.9%)	0.610
BoNT-A	34 (13.7%)		25 (13.1%)		9 (15.5%)	0.664
Ongoing preventive treatments						
Ongoing preventive treatment	180 (72.3%)	133 (69.6%)		47 (81.0%)		0.176
Anticonvulsants	25 (10.0%)	14 (7.3%)		11 (19.0%)		**0.022**
Beta-blockers	19 (7.6%)	16 (8.4%)		3 (5.2%)		0.576
Calcium channel blockers	2 (0.8%)	2 (1.0%)		0 (0.0%)		1.000
Sartans	7 (2.8%)	5 (2.6%)		2 (3.4%)		0.666
SSRI	10 (4.0%)	7 (3.7%)		3 (5.2%)		0.702
SNRI	7 (2.8%)	5 (2.6%)		2 (3.4%)		0.666
Tricyclic antidepressants	45 (18.1%)	31 (16.2%)		14 (24.1%)		0.177
BoNT-A	34 (13.7%)	28 (14.7%)		6 (10.3%)		0.515
Anti-CGRP mAbs	71 (28.5%)	53 (27.7%)		18 (31.0%)		0.622

*Legend and abbreviations: Values in bold are statistically significant. MIDAS, migraine disability assessment; HIT-6,headache impact test; CGRP, calcitonin gene related peptide; mAbs, monoclonal antibodies, BoNT-A, botulinumtoxin-A; SSRI, selective serotonin reuptake inhibitors; SNRI, serotonin-norepinephrine reuptake inhibitor*

### First treated attack

In the lasmiditan group, the majority of individuals took the 100 mg dose (41, 70.7%). There was no difference in the delay between attack onset and lasmiditan or rimegepant intake (timing of intake 53.9 [30.3–77.5] minutes for lasmiditan vs. 86.7 [66.1–107.3] minutes for rimegepant, p = 0.105), although a higher percentage of subjects took lasmiditan within one hour from headache onset (77.6% for lasmiditan vs. 58.1% for rimegepant, p = 0.008) (Table 2).

**Table 2:** Clinical features at drug intake in the first-treated migraine attack

	RIMEGEPANT	LASMIDITAN	p-value
Number of participants	**191**	**58**	-
Timing of drug intake			
Minutes from attack onset	86.7 [66.1–107.3]	53.9 [30.3–77.5]	0.105
Proportion of participants taking the drug more than 1 h from pain onset	80 (41.9%)	13 (22.4%)	**0.008**
Pain intensity			
Mild	9 (4.7%)	3 (5.2%)	0.987
Moderate	95 (49.7%)	29 (50.0%)	
Severe	87 (45.5%)	26 (44.8%)	
Disability level			
None	5 (2.6%)	2 (3.4%)	0.217
Mild	48 (25.1%)	7 (12.1%)	
Moderate/severe	103 (53.9%)	37 (63.8%)	
Complete	35 (18.3%)	12 (20.7%)	
Associated symptoms			
Photophobia	130 (68.1%)	46 (79.3%)	0.138
Phonophobia	115 (60.2%)	41 (70.7%)	0.165
Nausea	110 (57.6%)	41 (70.7%)	0.091
Vomiting	17 (8.9%)	3 (5.2%)	0.581
None	22 (11.5%)	3 (5.2%)	0.214
Most bothersome symptom			
Number of attacks available	162	57	0.580
Photophobia	46 (28.4%)	19 (33.3%)	
Phonophobia	36 (22.2%)	8 (14.0%)	
Nausea	76 (46.9%)	28 (49.1%)	
Vomiting	4 (2.5%)	2 (3.5%)	
Severity of the most bothersome symptom			
Number available	161	56	0.553
None	5 (3.1%)	1 (1.8%)	
Mild	35 (21.7%)	10 (17.9%)	
Moderate/severe	121 (75.1%)	45 (80.4%)	

*Legend and abbreviations: Values in bold are statistically significant. The number of patients included in the subgroup is reported where appropriate*

At the time of intake, most participants rated pain intensity as moderate-severe (95.2% for rimegepant and 94.8% for lasmiditan, p = 0.987). They also mainly reported a moderate-severe disability, with no differences between the two drugs (53.9% for rimegepant and 63.8% for lasmiditan, p = 0.217). Associated symptoms were represented mainly by photophobia and phonophobia, comparable for both cohorts. Similarly, both cohorts also indicated nausea as the most bothersome symptom (46.9% for rimegepant and 49.1% for lasmiditan, p = 0.580).

**Fig. 1:**
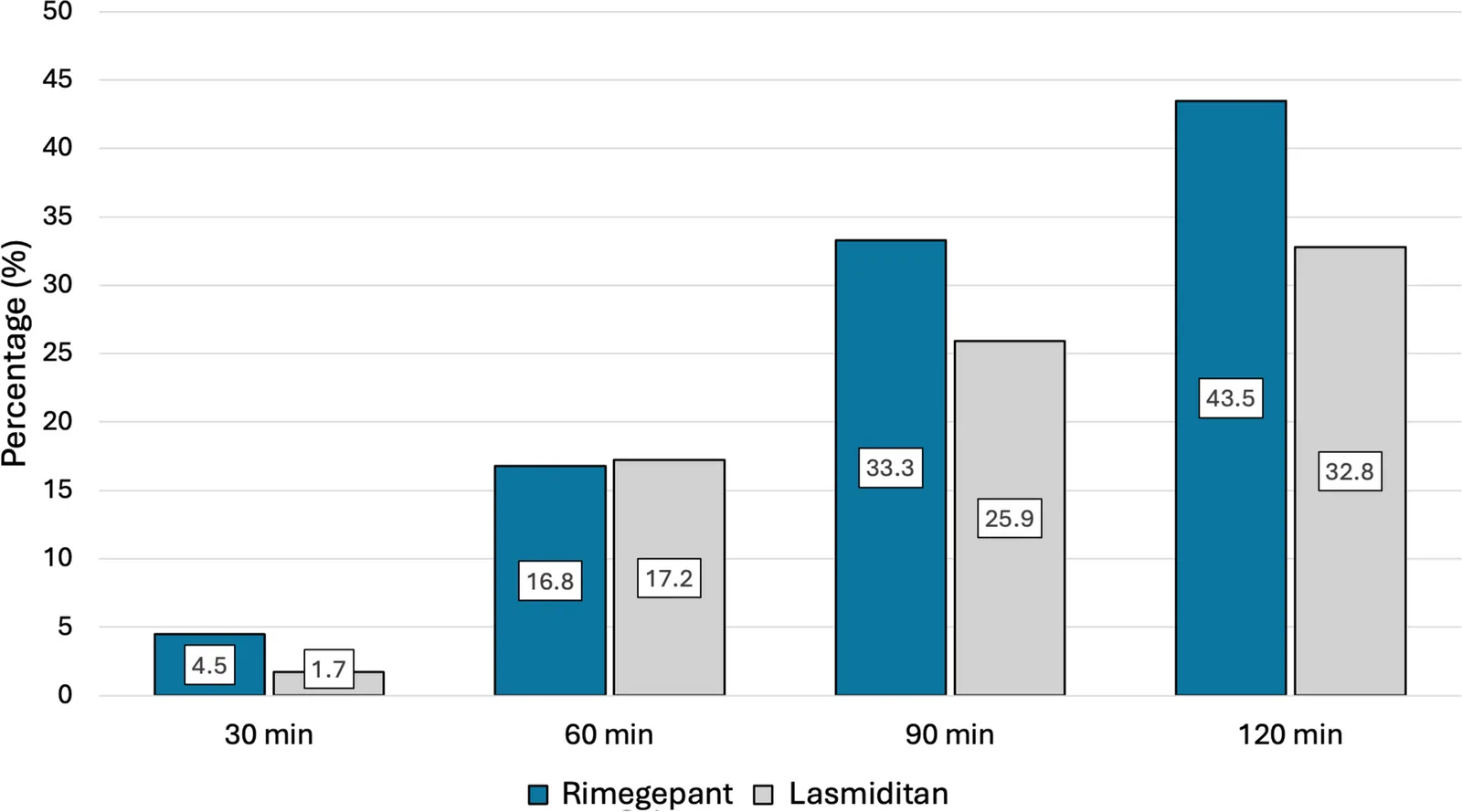
Pain freedom rates every 30 minutes up to 2 hours after rimegepant (blue) and lasmiditan (grey) intake

The proportion of participants achieving 2-hour pain freedom was comparable between rimegepant (43.5%, n = 83) and lasmiditan (32.8%, n = 19, p = 0.171) (Fig. 1 and Table 3).

**Table 3:** Comparative effectiveness and tolerability of rimegepant and lasmiditan in the first-treated attack

	RIMEGEPANT		LASMIDITAN		z-score	p-value
Effectiveness						
Number of participants	191		58	-		-
2-hour pain freedom	83 (43.5%)		19 (32.8%)	-		0.171
RR (lasmiditan vs. rimegepant)	0.75 [0.50–1.28]			1.375		0.169
Number needed to treat	9.35 [27.082 (harm) to ∞ to 3.986 (benefit)]			-		-
Tolerability						
Participants with at least one AE	22 (11.5%)	31 (53.4%)		-		**0.001**
RR (lasmiditan vs. rimegepant)	4.64 [2.93—7.35]			6.530		** < 0.001**
Number needed to harm	2.38 [3.22 (harm)—1.89 (harm)]			-		-

*Legend and abbreviations: Values in bold are statistically significant. AE: Adverse Events; RR: Relative Risk*

**Fig. 2:**
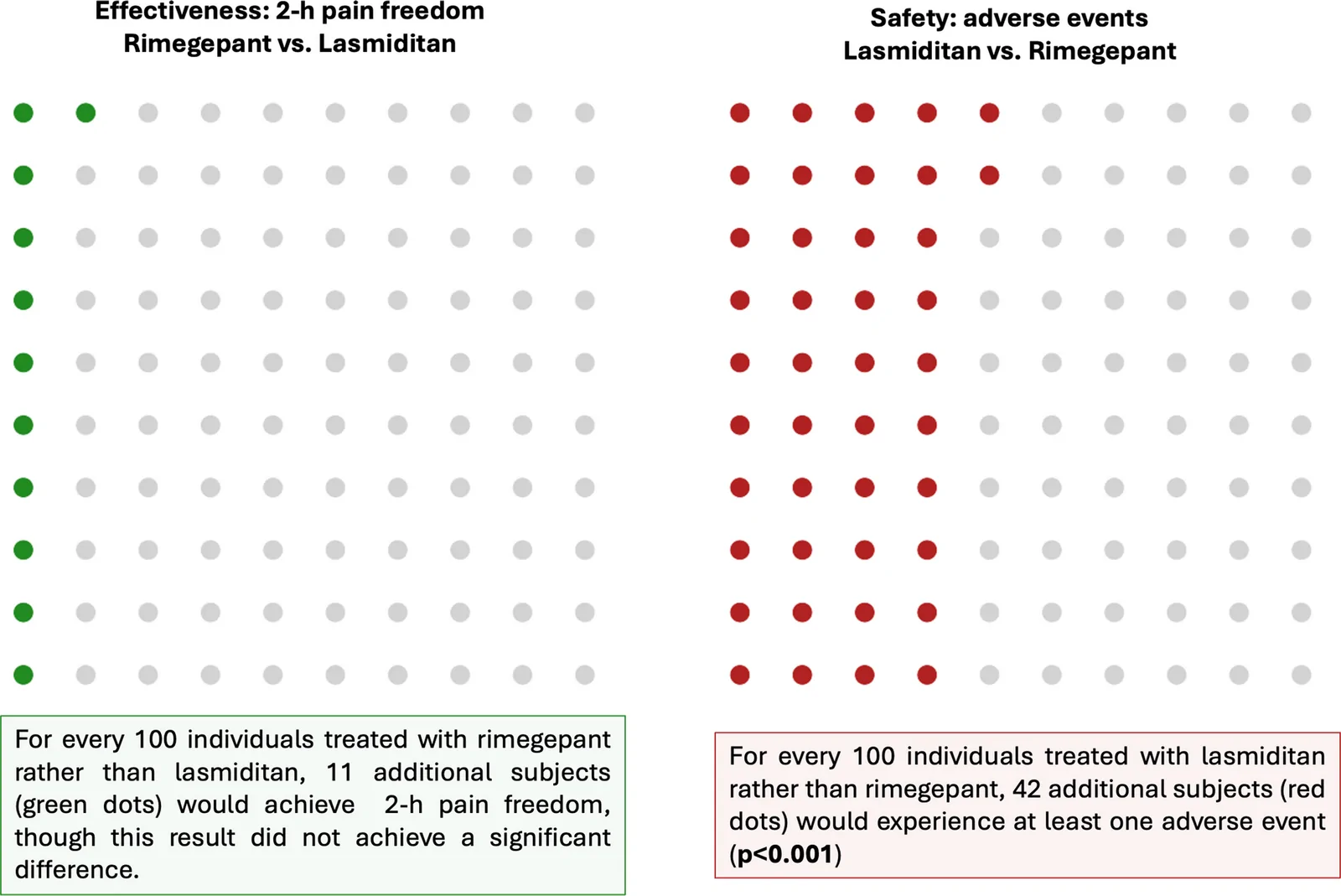
Graphical representation of number needed to treat and number needed to harm

The relative risk (RR) for 2-hour​​​ pain freedom for lasmiditan vs. rimegepant was 0.75 ([0.50–1.28], z = 1.375, p = 0.169), corresponding to a NNT of 9.35 [27.082 (harm) to ∞ to 3.986 (benefit)]. Based on the estimated absolute risk difference, for every 100 individuals treated with rimegepant rather than lasmiditan, approximately 11 additional subjects would achieve pain freedom at 2 hours. However, it should be interpreted with caution as the treatment difference was not statistically significant (Fig. 2).

The small proportion of participants achieving 2-hour pain freedom at 30 minutes progressively increased over the subsequent 30-minute intervals post dose for both drugs (Supplementary Table 2). Rimegepant group exhibited a lower 2-hour disability (no disability 2-hour post dose: rimegepant 47.9% vs. lasmiditan 24.1%, p = 0.013) and a lower 2-hour MBS severity (no MBS 2-hour post dose: 50.0% for rimegepant vs. 39.7% for lasmiditan, p = 0.036), without differences in the complete resolution of MBS at 2 hours (p = 0.096).

The percentage of participants with at least one AE was 11.5% (22/191) after rimegepant intake and 53.4% (31/58) after lasmiditan intake (p = 0.001) (Table 3). All adverse events were mild, and self-limiting. The RR for experiencing at least one AE for lasmiditan vs. rimegepant was 4.64 [2.93–7.35], z = 6.530; p < 0.001), corresponding to a NNH of 2.38 [3.22 (harm)—1.89 (harm)]. This means that for every 100 individuals treated with lasmiditan rather than rimegepant, 42 additional subjects would experience at least one adverse event (Fig. 2).

### All-treated migraine attacks

In the GAINER cohort, participants overall treated 377 attacks with rimegepant (191 one, 110 two, 46 three and 30 four). In the DART cohort, participants treated 100 attacks with lasmiditan (58 one, 28 two, 9 three and 5 four), with the 100 mg dosage being the most used (n = 75, 75%).

Lasmiditan was taken earlier than rimegepant: the proportion of attacks treated within one hour from pain onset was higher for lasmiditan (79%, n = 79) than for rimegepant (57.8%, n = 218, p = 0.001). In the lasmiditan cohort, associated symptoms were present in a higher percentage of attacks (97% for lasmiditan vs. 84.4% for rimegepant, p = 0.001), mainly represented by photophobia (79% for lasmiditan vs. 67.4% for rimegepant, p = 0.027) and nausea (72.0% for lasmiditan vs. 53.8% for rimegepant, p = 0.001). Detailed description of baseline features of all-treated attacks is reported in Table 4.

**Table 4:** Clinical features at drug intake in all treated attacks

		**RIMEGEPANT**	**LASMIDITAN**	**p-**value
Number of participants		191	58	-
Number of treated attacks	One	191 (50.7%)	58 (58.0%)	0.476
	Two	110 (29.2%)	28 (28.0%)	
	Three	46 (12.2%)	9 (9.0%)	
	Four	30 (8.0%)	5 (5.0%)	
	Total	**377**	**100**	
Pain intensity				
Mild		36 (9.5%)	5 (5.0%)	0.208
Moderate		181 (48.0%)	56 (56.0%)	
Severe		160 (42.5%)	39 (39.0%)	
Timing of intake				
Minutes from attack onset		77.2 [64.6–89.7]	53.1 [35.6–70.5]	0.068
Proportion of attacks in which the drug was taken after 1 hour from pain onset		159 (42.2%)	21 (21.0%)	**0.001**
Disability level				
None		17 (4.5%)	3 (3.0%)	0.102
Mild		86 (22.8%)	13 (13.0%)	
Moderate/severe		203 (53.8%)	66 (66.0%)	
Complete		71 (18.8%)	18 (18.0%)	
Associated symptoms				
Photophobia		254 (67.4%)	79 (79.0%)	**0.027**
Phonophobia		223 (59.2%)	69 (69.0%)	0.083
Nausea		203 (53.8%)	72 (72.0%)	**0.001**
Vomiting		24 (6.4%)	8 (8.0%)	0.509
None		59 (15.6%)	3 (3.0%)	**0.001**
Most bothersome symptom				
Number of attacks available		311	99	0.648
Photophobia		97 (31.2%)	34 (34.3%)	
Phonophobia		67 (21.5%)	10 (10.1%)	
Nausea		141 (45.3%)	51 (51.5%)	
Vomiting		6 (1.9%)	4 (4.0%)	
Severity of most bothersome symptom				
Number available		310	98	0.758
None		8 (2.6%)	3 (3.1%)	
Mild		66 (21.3%)	18 (18.4%)	
Moderate/severe		236 (76.1%)	77 (78.6%)	

*Legend: Values in bold are statistically significant. The number of patients included in the subgroup is reported where appropriate*

The proportion of participants achieving 2-hour pain freedom in all treated attacks was comparable between rimegepant (44.6%, n = 168) and lasmiditan (37.0%, n = 37, p = 0.211) (Table 5). The RR was 0.83 ([0.63–1.10]; z = 1.304, p = 0.192), corresponding to a NNT of 13.22 [30.02 (harm) to ∞ to 5.42 (benefit)].

**Table 5:** Comparative effectiveness and tolerability of rimegepant and lasmiditan in all treated attacks

	RIMEGEPANT		LASMIDITAN	z-score	p-value
Effectiveness					
Number of attacks	377		100	-	-
2-h pain freedom	168 (44.6%)		37 (37.0%)	-	0.211
RR (lasmiditan vs. rimegepant)	0.83 [0.63–1.10]			1.304	0.192
Number needed to treat	13.22 [30.02 (harm) to ∞ to 5.42 (benefit)]			-	-
Tolerability					
Attacks with at least one AE	29 (7.7%)	42 (42.0%)		-	**0.001**
RR (lasmiditan vs. rimegepant)	5.46 [3.59–8.30]			7.946	** < 0.001**
Number needed to harm	2.92 [3.69 (harm) to 2.41 (harm)]			-	-

*Legend and abbreviations: Values in bold are statistically significant. AE: Adverse Events; RR: Relative Risk*

When compared to lasmiditan, rimegepant presented a lower 2-hour disability (no disability 2-hour post dose: 49.7% for rimegepant vs. 31% for lasmiditan, p = 0.006) and a lower 2-hour MBS severity (no MBS 2-hour post dose: 56.4% for rimegepant vs. 45.0% for lasmiditan, p = 0.002). The rimegepant cohort had a lower prevalence of 2-hour post dose photophobia (23.9% vs. 38.0%, p = 0.007), phonophobia (19.4% vs. 33% p = 0.006), and nausea (20.2% vs. 32.0%, p = 0.015) (Supplementary Table 3).

For all treated attacks, we considered the number of attacks with at least one AE; the RR was 5.46 [3.59–8.30]; z = 7.946, p < 0.001), corresponding to a NNH of 2.92 [3.69 (harm) to 2.41 (harm)].

The most prevalent AEs reported in all-treated attacks in the rimegepant cohort were fatigue (1.9%, n = 7/377), gastrointestinal symptoms (1.9%, n = 7/377), somnolence (1.3%, n = 5/377), and transient cognitive difficulties (1.1%, n = 4/377). In the lasmiditan cohort, the most common AEs (> 5%) reported for all-treated attacks were asthenia (20.0%, n = 20/100), dizziness (16.0%, n = 16/100), somnolence (11%, n = 11/100), anxiety or agitation (5%, n = 5/100), and paraesthesia (7%, n = 7/100).

## Discussion

In this study we provide an indirect comparison of the effectiveness and tolerability of rimegepant and lasmiditan for the acute treatment of migraine attacks, offering a first evidence in the real-world setting. We used data collected in two individual real-world studies (GAINER and DART) sharing the core study procedures, inclusion/exclusion criteria, outcomes, centres involved and methods for data collection, as demonstrated by the comparable clinical and demographic features of the enrolled populations [8, 30]. The pooled population presented in this paper included 249 individuals, of whom 58 took lasmiditan for treating 100 attacks and 191 took rimegepant for a total of 377 attacks treated. The only differences that emerged between the two individual cohorts are the higher number of previously failed preventive treatment classes and the higher rate of baseline associated symptoms among participants taking lasmiditan, potentially suggesting a more treatment-resistant cohort, despite similar baseline headache frequency and disability levels.

Our findings can be summarized as follows: i) the proportion of participants achieving 2-hour pain freedom was comparable between rimegepant and lasmiditan cohorts for both first-treated and all-treated attacks with a non-significant relative risk for achieving 2-hour pain freedom; ii) the proportion of participants with at least one AE was higher after lasmiditan intake compared to rimegepant, and for every 2 subjects treated with lasmiditan instead of rimegepant one additional subject would experience at least one AE (NNH = 2.38).

In line with our study, multiple previous RCTs and real-world evidence have demonstrated a higher efficacy over placebo and good effectiveness in clinical practice of both drugs [8, 10–16, 30]. Indirect comparisons of published RCTs yielded inconsistent results. Some analyses found a comparable efficacy [26, 27], whereas others detected a slightly higher rate of 2-hour pain freedom for lasmiditan, especially for the 200 mg dose, compared with rimegepant (OR of 2-hour pain freedom: lasmiditan 200 mg = 2.88 [2.22–3.73] vs. lasmiditan 100 mg = 2.28 [1.75–2.96] vs. rimegepant 75 mg = 2.00 [1.45–2.75]) [28, 29]. Our findings align with the indirect comparative analyses that did not detect differences in the effectiveness of the two drugs, despite the obvious differences in the setting. Indeed, in our two real-world studies, we adopted broader inclusion criteria, enrolling both chronic and episodic migraine individuals, and applied less strict treatment indications. Participants were allowed to take the acute medication at their preferred timepoint after headache onset, including when pain was still mild. Furthermore, lasmiditan dosage was left to the prescriber’s discretion, with most participants receiving the 100 mg dose. 

Regarding safety and tolerability, lasmiditan presented a significantly higher rate of AEs. These findings are in line with previous indirect comparisons from RCTs, which demonstrated a higher incidence of AEs with lasmiditan than with rimegepant [26–29].

According to our analyses, for every 100 individuals treated with lasmiditan instead of rimegepant, nearly 42 additional subjects would experience at least one AE. The high RR of AEs associated with lasmiditan is consistent with previous post-hoc analyses from the SAMURAI and SPARTAN studies, as well as the CENTURION trial [28, 36, 37]. Indirect comparisons from these RCTs reported a cumulative odds ratio of AEs equal to 3.64 for the 100 mg dose [28], with a dose-related emergence of AEs [27].

A recent probabilistic model study using data coming from 12 clinical trials compared the cost of rimegepant and lasmiditan associated AEs. This model suggested that prescription of rimegepant compared to lasmiditan would result in a saving of around €600/patient every 6 months [38].

Considering that the 2-hour pain freedom rate achieved with currently available acute migraine treatments ranges from approximately 10% to 40% [29], and that a substantial proportion of subjects either do not respond to, or have contraindications to, certain therapeutic classes, lasmiditan represented an important additional treatment option for a subset of migraine individuals with unmet therapeutic needs. Unfortunately, the commercial production of lasmiditan has been discontinued [39].

Its discontinuation may have been influenced in part by its unfavorable tolerability profile, particularly the high frequency of central nervous system AEs such as dizziness and somnolence. However, an even greater impact was likely exerted by the mandatory recommendation to refrain from driving or operating machinery for at least 8 hours after dosing [40]. This restriction was based on dedicated driving simulation studies demonstrating impaired driving performance following lasmiditan administration [41]. However, similar driving assessments have not been systematically performed for many other acute migraine treatments or analgesics that may also impair psychomotor performance, making direct comparisons difficult and raising questions about the consistency of regulatory requirements across therapeutic classes.

We must acknowledge some limitations of our post-hoc analysis. First, our results are limited by the indirect nature of the comparison, without a solid head-to-head study design. Furthermore, we did not perform a comparison across different lasmiditan dosages, as the dosage was prescribed at the physician’s discretion, in accordance with the real-world study design. The resulting unbalanced distribution of participants across dosage groups precluded proper statistical analyses. Second, the presence of differences in the baseline study population, though minor, should be taken into account as a limitation in the final comparison. Third, a formal sample size calculation was not performed, although a relatively large population was enrolled in both studies. Fourth, the absence of reimbursement criteria, together with the requirement for a neurologist’s prescription to obtain lasmiditan or rimegepant, may have introduced a potential selection bias. Indeed, we may have inadvertently enrolled a difficult-to-treat population more likely to seek specialist medical care. Finally, we were unable to perform a comprehensive analysis of effectiveness and tolerability in individuals with cardiovascular or psychiatric comorbidities, as these coexisting conditions were not systematically collected in sufficient detail to allow a thorough identification.

## Conclusion

This real-world post-hoc study provided an indirect comparison between lasmiditan and rimegepant in the acute treatment of migraine attacks. Both drugs showed a good and similar effectiveness, while lasmiditan was associated with a higher rate of adverse events. Accordingly, rimegepant emerges as the preferred option in this indirect comparison, combining comparable effectiveness with a more favorable tolerability profile, a key determinant of treatment success and patient satisfaction.

Nevertheless, regardless of the reasons underlying its commercial discontinuation, we believe that the withdrawal of lasmiditan represents a setback for precision medicine and a loss for individuals with limited treatment alternatives, including those with contraindications to triptans or an inadequate response to available acute therapies. As migraine management increasingly moves toward a personalized approach, maintaining a diverse therapeutic armamentarium remains essential to address the heterogeneity of patients' needs and preserve access to effective treatments, even when they are intended for relatively small but clinically meaningful patient populations.

## Supplementary Information

Below is the link to the electronic supplementary material.

Supplementary file1 (DOCX 4579 KB)

## Data Availability

Data supporting the findings reported in the present study are illustrated in the manuscript and in the supplementary materials. The data collected and analyzed for the current study are available from the corresponding author on reasonable request.
