# Supplementary material for: Effectiveness and safety of lasmiditan and rimegepant in the acute migraine treatment: an indirect real-world comparison
Source: Neurol Sci. 2026 Nov 5;47(11):917. doi: 10.1007/s10072-026-09510-6 (PMC13736438; doi:10.1007/s10072-026-09510-6)
Supplement: Supplementary file 1 [file 10072_2026_9510_MOESM1_ESM.docx]

**Supplementary materials**

**Effectiveness and safety of lasmiditan and rimegepant in the acute migraine treatment: an indirect real-world comparison**

**Methods**

**Table 1. Centers involved in the second analysis of the GAINER and DART studies**

_______________________________________________________________________________

1 Department of Brain and Behavioral Sciences, University of Pavia, Pavia, Italy.

2 Headache Science and Neurorehabilitation Unit, IRCCS Mondino Foundation, Pavia, Italy

3 Digital and Predictive Medicine, Pharmacology and Clinical Metabolic Toxicology-Headache Center and Drug Abuse-Laboratory of Clinical Pharmacology and Pharmacogenomics, AOU Policlinico di Modena, Modena, Italy;

4 Department of Medico-Surgical Sciences and Biotechnologies, Polo Pontino, Sapienza University of Rome, C.so della Repubblica 79, 04100, Latina, Italy.

5 Department of Neurosciences “Rita Levi Montalcini”, University of Turin, Turin, Italy

6 Fondazione Policlinico Universitario Agostino Gemelli IRCCS, Università Cattolica del Sacro Cuore, Rome, Italy

7 Department of Biotechnological and Applied Clinical Sciences, University of L’Aquila, 67100, L’Aquila, Italy

8 Department of Health Sciences, University of Florence, Florence, Italy

9 Headache Center, Department of Medical, Surgical, Neurological, Metabolic and Aging Sciences, University of Campania ‘Luigi Vanvitelli’ Naples, Italy

10 Headache Center of Clinical Neurology of Istituto Clinico Città Di Brescia, Brescia, Italy.

11 Neuroalgology Unit and Headache Center, Fondazione IRCCS Istituto Neurologico Carlo Besta, Milan, Italy

12 L. Amaducci” Neurological Clinic, University of Bari, Bari, Italy

13 IRCCS Istituto delle Scienze Neurologiche di Bologna, UOC Clinica Neurologica Metropolitana - Programma Cefalee ed Algie Facciali.

14 Neurology Unit, Ospedale Santo Stefano, USL Toscana Centro, Prato, Italy

15 Headache Centre, Neurology - Euganea, Health Unit, Padua, Padua, Italy

16 Clinical Neurology Azienda Sanitaria Friuli Centrale. Department of Medicine University of Udine, Udine, Italy

17 Department of Medical, Surgical and Neurological Sciences, University of Siena, Siena, Italy

18 Headache Centre, Clinical Unit of Neurology, School of Neurology, Department of Medicine, Surgery and Health Sciences, University Hospital and Health Services, ASUGI, University of Trieste, Trieste, Italy.

19 Unit of Neurology, Department of Systems Medicine, University of Rome Tor Vergata

20 Department of Neurosciences, AOU Renato Dulbecco, Catanzaro, Italy

21 Department of Neurology- Laboratory of Neuroscience, IRCCS Istituto Auxologico Italiano, Milan, Italy

**Supplementary table 2. Effectiveness parameters collected every 30 minutes up to 2-hour post dose for the first-treated attack**

|  | **Rimegepant** | **Lasmiditan** | **p-value** |
| --- | --- | --- | --- |
| **N** | **191** | **58** | - |
| **Pain intensity at 30 minutes** | | | |
| **Number of attack available** | 179 | 58 | 0.460 |
| **Pain-free** | 8 (4.5%) | 1 (1.7%) |  |
| **Mild** | 43 (24.0%) | 17 (29.3%) |  |
| **Moderate** | 75 (41.9%) | 24 (41.4%) |  |
| **Severe** | 53 (29.6%) | 16 (27.6%) |  |
| **Pain intensity at 60 minutes** | | | |
| **Number of attack available** | 179 | 58 | 1.000 |
| **Pain-free** | 30 (16.8%) | 10 (17.2%) |  |
| **Mild** | 54 (30.2%) | 13 (22.4%) |  |
| **Moderate** | 68 (38.0%) | 26 (44.8%) |  |
| **Severe** | 27 (15.1%) | 9 (15.5%) |  |
| **Pain intensity at 90 minutes** | | | |
| **Number of attack available** | 177 | 58 | 0.331 |
| **Pain-free** | 59 (33.3%) | 15 (25.9%) |  |
| **Mild** | 60 (33.9%) | 19 (32.8%) |  |
| **Moderate** | 36 (20.3%) | 19 (32.8%) |  |
| **Severe** | 22 (12.4%) | 5 (8.6%) |  |
| **Pain intensity at 2 hours** | | | |
| **Pain-free** | 83 (43.5%) | 19 (32.8%) | 0.171 |
| **Mild** | 50 (26.2%) | 21 (36.2%) |  |
| **Moderate** | 43 (22.5%) | 14 (24.1%) |  |
| **Severe** | 15 (7.9%) | 4 (6.9%) |  |
| **Disability level at 2 hours** | | | |
| **Number of attack available** | 190 | 58 | **0.013** |
| **None** | 91 (47.9%) | 14 (24.1%) |  |
| **Mild** | 50 (26.3%) | 25 (43.1%) |  |
| **Moderate/severe** | 36 (18.9%) | 14 (24.1%) |  |
| **Complete** | 13 (6.8%) | 5 (8.6%) |  |
| **Associated symptoms at 2 hours** | | | |
| **Photophobia** | 51 (26.7%) | 23 (39.7%) | 0.071 |
| **Phonophobia** | 37 (19.4%) | 20 (34.5%) | **0.021** |
| **Nausea** | 43 (22.5%) | 19 (32.8%) | 0.121 |
| **Vomiting** | 4 (2.1%) | 2 (3.4%) | 0.626 |
| **None** | 99 (51.8%) | 23 (39.7%) | 0.133 |
| **Most bothersome symptom severity at 2 hours** | | | |
| **Number of attack available** | 166 | 58 |  |
| **None** | 83 (50.0%) | 23 (39.7%) | **0.036** |
| **Mild** | 49 (29.5%) | 14 (24.1%) |  |
| **Moderate/severe** | 34 (20.5%) | 21 (36.2%) |  |
| **24-hour follow-up** | | | |
| **Use of rescue medication** | 44 (23.2%) n=190 | 13 (22.4%)  n=58 | 1.000 |
| **Pain freedom at 24 h** | 106 (62.0%) n=171 | 33 (56.9%)  n=58 | 0.636 |
| **No pain relapse 2 h – 24 h** | 69 (40.4%) n=171 | 18 (31.0%)  n=58 | 0.108 |

*Legend: Values in bold are statistically significant. The number of patients included in the subgroup is reported where appropriate.*

**Supplementary table 3. Effectiveness parameters collected every 30 minutes up to 2-hour post dose for all-treated attacks**

|  | **Rimegepant** | **Lasmisditan** | **p-value** |
| --- | --- | --- | --- |
| **Number of participants** | 191 | 58 | - |
| **Total attacks** | 377 | 100 | - |
| **Pain intensity at 30 minutes** | | | |
| **Number of attacks available** | 360 | 100 | 1.000 |
| **Pain-free** | 21 (5.8%) | 5 (5.0%) |  |
| **Mild** | 99 (27.5%) | 28 (28.0%) |  |
| **Moderate** | 135 (37.5%) | 42 (42.0%) |  |
| **Severe** | 105 (29.2%) | 25 (25.0%) |  |
| **Pain intensity at 60 minutes** | | | |
| **Number of attacks available** | 360 | 100 | 1.000 |
| **Pain-free** | 71 (19.7%) | 20 (20.0%) |  |
| **Mild** | 112 (31.1%) | 21 (21.0%) |  |
| **Moderate** | 122 (33.9%) | 45 (45.0%) |  |
| **Severe** | 55 (15.3%) | 14 (14.0%) |  |
| **Pain intensity at 90 minutes** | | | |
| **Pain-free** | 131 (36.5%) | 30 (30.0%) | 0.239 |
| **Mild** | 110 (30.6%) | 31 (31.0%) |  |
| **Moderate** | 77 (21.4%) | 31 (31.0%) |  |
| **Severe** | 41 (11.4%) | 8 (8.0%) |  |
| **Pain intensity at 2 hours** | | | |
| **Pain-free** | 168 (44.6%) | 37 (37.0%) | 0.211 |
| **Mild** | 97 (25.7%) | 32 (32.0%) |  |
| **Moderate** | 86 (22.8%) | 25 (25.0%) |  |
| **Severe** | 26 (6.9%) | 6 (6.0%) |  |
| **Disability level at 2 hours** | | | |
| **Number of attacks available** | 372 | 100 | **0.006** |
| **None** | 185 (49.7%) | 31 (31.0%) |  |
| **Mild** | 95 (25.5%) | 37 (37.0%) |  |
| **Moderate/severe** | 70 (18.8%) | 27 (27.0%) |  |
| **Complete** | 22 (5.9%) | 5 (5.0%) |  |
| **Associated symptoms at 2 hours** | | | |
| **Photophobia** | 90 (23.9%) | 38 (38.0%) | **0.007** |
| **Phonophobia** | 73 (19.4%) | 33 (33.0%) | **0.006** |
| **Nausea** | 76 (20.2%) | 32 (32.0%) | **0.015** |
| **Vomiting** | 6 (1.6%) | 3 (3.0%) | 0.405 |
| **None** | 219 (58.1%) | 45 (45.0%) | **0.023** |
| **Severity of most bothersome symptom at 2 hours** | | | |
| **Number of attacks available** | 344 | 100 | **0.002** |
| **None** | 194 (56.4%) | 45 (45.0%) |  |
| **Mild** | 85 (24.7%) | 22 (22.0%) |  |
| **Moderate/severe** | 65 (18.9%) | 33 (33.0%) |  |

*Legend: Values in bold are statistically significant.*
